# Drugs for treating infections caused by non-tubercular mycobacteria: a narrative review from the study group on mycobacteria of the Italian Society of Infectious Diseases and Tropical Medicine

**DOI:** 10.1007/s15010-024-02183-3

**Published:** 2024-02-08

**Authors:** A. Calcagno, N. Coppola, L. Sarmati, M. Tadolini, R. Parrella, A. Matteelli, N. Riccardi, M. Trezzi, A. Di Biagio, V. Pirriatore, A. Russo, G. Gualano, E. Pontali, L. Surace, E. Falbo, J. Mencarini, F. Palmieri, A. Gori, M. Schiuma, G. Lapadula, D. Goletti, for the Study Group on Mycobacteria (MYGRO) of the Italian Society of Infectious Diseases and Tropical Medicine (SIMIT).

**Affiliations:** 1 Unit of Infectious Diseases, Department of Medical Sciences, University of Turin, Turin, Italy; 2 Stop TB Italy, Milan, Italy; 3 Infectious Diseases Unit, Section of Infectious Diseases, Department of Mental Health and Public Medicine, University of Campania Luigi Vanvitelli, Naples, Italy; 4 Department of System Medicine, Tor Vergata University and Infectious Disease Clinic, Policlinico Tor Vergata, Rome, Italy; 5 Infectious Diseases Unit, IRCCS Azienda Ospedaliero-Universitaria Di Bologna, Bologna, Italy; 6 Department of Medical and Surgical Sciences, Alma Mater Studiorum University of Bologna, Bologna, Italy; 7 Respiratory Infectious Diseases Unit, Cotugno Hospital, A. O. R. N. dei Colli, Naples, Italy; 8 Institute of Infectious and Tropical Diseases, WHO Collaborating Centre for TB Prevention, Department of Clinical and Experimental Sciences, University of Brescia, Brescia, Italy; 9 Infectious Diseases Unit, Department of Clinical and Experimental Medicine, Azienda Ospedaliero Universitaria Pisana, University of Pisa, Pisa, Italy; 10 Infectious and Tropical Diseases Unit, Department of Medical Sciences, Azienda Ospedaliero-Universitaria Senese, Siena, Italy; 11 Infectious Diseases Unit, San Martino Policlinico Hospital-IRCCS for Oncology and Neurosciences, Genoa, Italy; 12 Department of Health Sciences (DISSAL), University of Genoa, Genoa, Italy; 13 Unit of Infectious Diseases, “DivisioneA”, Ospedale Amedeo di Savoia, ASL CIttà di Torino, Turin, Italy; 14 Respiratory Infectious Diseases Unit, National Institute for Infectious Diseases Lazzaro Spallanzani-IRCCS, Rome, Italy; 15 Department of Infectious Diseases, Galliera Hospital, Genoa, Italy; 16 Dipartimento Di Prevenzione, Azienda Sanitaria Provinciale di Catanzaro, Centro di Medicina del Viaggiatore e delle Migrazioni, P. O. Giovanni Paolo II, Lamezia Terme, CZ Italy; 17 Infectious and Tropical Diseases Unit, Careggi University Hospital, Florence, Italy; 18 Dipartimento di Fisiopatologia Medico-Chirurgica e dei Trapianti, ASST Fatebenefratelli Sacco-Ospedale Luigi Sacco-Polo Universitario and Università Degli Studi di Milano, Milano, Italy; 19 Infectious Diseases Unit, Fondazione IRCCS San Gerardo dei Tintori, University of Milano-Bicocca, Monza, Italy; 20 Translational Research Unit, Epidemiology Department, National Institute for Infectious Diseases-IRCCS L. Spallanzani, Rome, Italy

**Keywords:** NTM, Pharmacology, Side effects, Clofazimine, Therapy

## Abstract

**Background:**

Non-tuberculous mycobacteria (NTM) are generally free-living organism, widely distributed in the environment, with sporadic potential to infect. In recent years, there has been a significant increase in the global incidence of NTM-related disease, spanning across all continents and an increased mortality after the diagnosis has been reported. The decisions on whether to treat or not and which drugs to use are complex and require a multidisciplinary approach as well as patients’ involvement in the decision process.

**Methods and Results:**

This review aims at describing the drugs used for treating NTM-associated diseases emphasizing the efficacy, tolerability, optimization strategies as well as possible drugs that might be used in case of intolerance or resistance. We also reviewed data on newer compounds highlighting the lack of randomised clinical trials for many drugs but also encouraging preliminary data for others. We also focused on non-pharmacological interventions that need to be adopted during care of individuals with NTM-associated diseases

**Conclusions:**

Despite insufficient efficacy and poor tolerability this review emphasizes the improvement in patients’ care and the needs for future studies in the field of anti-NTM treatments.

**Supplementary Information:**

The online version contains supplementary material available at 10.1007/s15010-024-02183-3.

## Introduction and methods

Non-tuberculous mycobacteria (NTM) are a group of free-living mycobacteria that can cause a wide spectrum of diseases in humans. Given the increasing incidence of NTM infections and the challenges health care workers encounter in treating them, a review of the available literature on the anti-NTM treatment strategies has been performed. We performed a comprehensive systematic search of articles published in peer reviewed journals using PubMed/MEDLINE (from 1980 until 2022). Reference lists of included papers were hand searched for additional relevant studies. The search was restricted to articles in English language.

## Epidemiology and risk factors

Non-tuberculous mycobacteria (NTM) are generally free-living organism, widely distributed in the environment, with sporadic potential to infect humans and cause non-tuberculous mycobacterial disease [1]. Slow-growing mycobacteria (SGM), such as *Mycobacterium avium* complex (MAC), are the most common strains associated with human disease, but this varies depending on factors, such as regional differences, patients’ characteristics, and anatomical site of infection [2–6]. Supplementary Table 1 reports studies evaluating the epidemiological, microbiological, and clinical characteristics of NTM-infections in different countries.

In recent years, there has been a significant increase in the global incidence of NTM-related disease, spanning across all continents [7]. Within the United States, two separate studies showed an increase in incidence of NTM infection, although different in magnitude. Specifically, the first study observed an increase in reported cases from 8.7/100,000 inhabitants in 2008 to 13.9/100,000 in 2013; in the second one, incidence progressed from 3.13/100,000 in 2008 to 4.73/100,000 in 2015 [8, 9]. Similar data were reported in multiple studies conducted in Europe and Asia. For instance, in Denmark, the incidence of NTM-related diseases increased from 1.3/100,000 in 2013 to 2.5/100,000 in 2021 [10–13]. Moreover, recent reports highlight a concerning increase in the prevalence of *Mycobacterium abscessus* (Mabs), a rapidly growing and hard-to-treat NTM [14].

Although considered less virulent than *Mycobacterium tuberculosis*, NTM can cause infections that affect various organ systems, with the lungs, skin, soft tissues, and lymph nodes being the most frequently involved [15]. NTM diseases predominantly affect subjects with anatomic or structural airways/lungs abnormalities, such as bronchiectasis, chronic obstructive pulmonary disease (COPD) and cystic fibrosis (CF), or those with immune-deficiency condition, such as HIV infection, solid organ transplant, and cancer [16–19]. Additionally, increased incidence in individuals with other comorbidities, such as dyslipidemia, diabetes mellitus, asthma, and gastro-oesophageal reflux disease (GERD) has also been reported [16].

## Diagnosis of NTM-associated diseases

The diagnosis of NTM disease is laborious and often challenging. The inherent nature of NTM as environmental microorganisms introduces the potential for their presence in biological samples due to contamination or colonization rather than true infection. Guidelines for NTM pulmonary disease (NTM-PD) establish three main criteria for diagnosis: clinical, radiological, and microbiological ones [20]. Clinical and radiological criteria comprise the presence of respiratory or systemic symptoms (low-grade fever, weight loss) coupled with radiological evidence of nodular or cavitary opacities using standard radiography or evidence of bronchiectasis surrounded by small nodules, as observed in high-resolution computed tomography. Microbiological criteria encompass positive culture results from either ≥2 sputum samples or a bronchial washing or histological evidence of mycobacterial invasion (such as granulomatous inflammation or the presence of acid-fast bacilli) coupled with positive culture results from lung tissue or from other respiratory samples.

No shared guidelines exist for establishing diagnostic criteria in cases of disseminated disease or NTM infection affecting sites other than the lung (e.g., skin, bones, muscles, and lymph nodes). Histological suspicion typically arises from tissue samples obtained from biopsy or surgical interventions, with definitive microbiological diagnosis confirmed by culture isolation and/or real-time polymerase chain reaction (PCR) detection, which allows for more rapid diagnosis [21–24].

The therapeutic approach to NTM infections is based on combined antibiotic regimens, owing to the natural drug resistance of some NTMs and the potential emergence of resistance during treatment. In selected cases, drug sensitivity testing may contribute to the selection of optimal medical treatments to achieve the most favorable therapeutic outcome. The Clinical & Laboratory Standard Institute (CLSI) recommends the broth microdilution test (Culture Species Identification Drug Susceptibility Testing-DST) to evaluate drug susceptibility of NTM isolates. In recent years, some drug resistance genes have also been identified (*rrl* and *erm* for macrolide resistance, and *rrs* for aminoglycoside resistance), and some comparative studies suggest a good performance of genotypic resistance tests, at least for some NTM species (*MAC, Mabs*). The use of efficient genotypic tests would overcome some limitations of phenotypic tests (e.g., long incubation times, antibiotic stability problems, and uniformity in the interpretation of results) and, in the near future, the use of the combination of phenotypic and genotypic tests will allow a better definition of drug susceptibility, at least for some NTM species [25].

## Non-pharmacological interventions

**Fig. 1:**
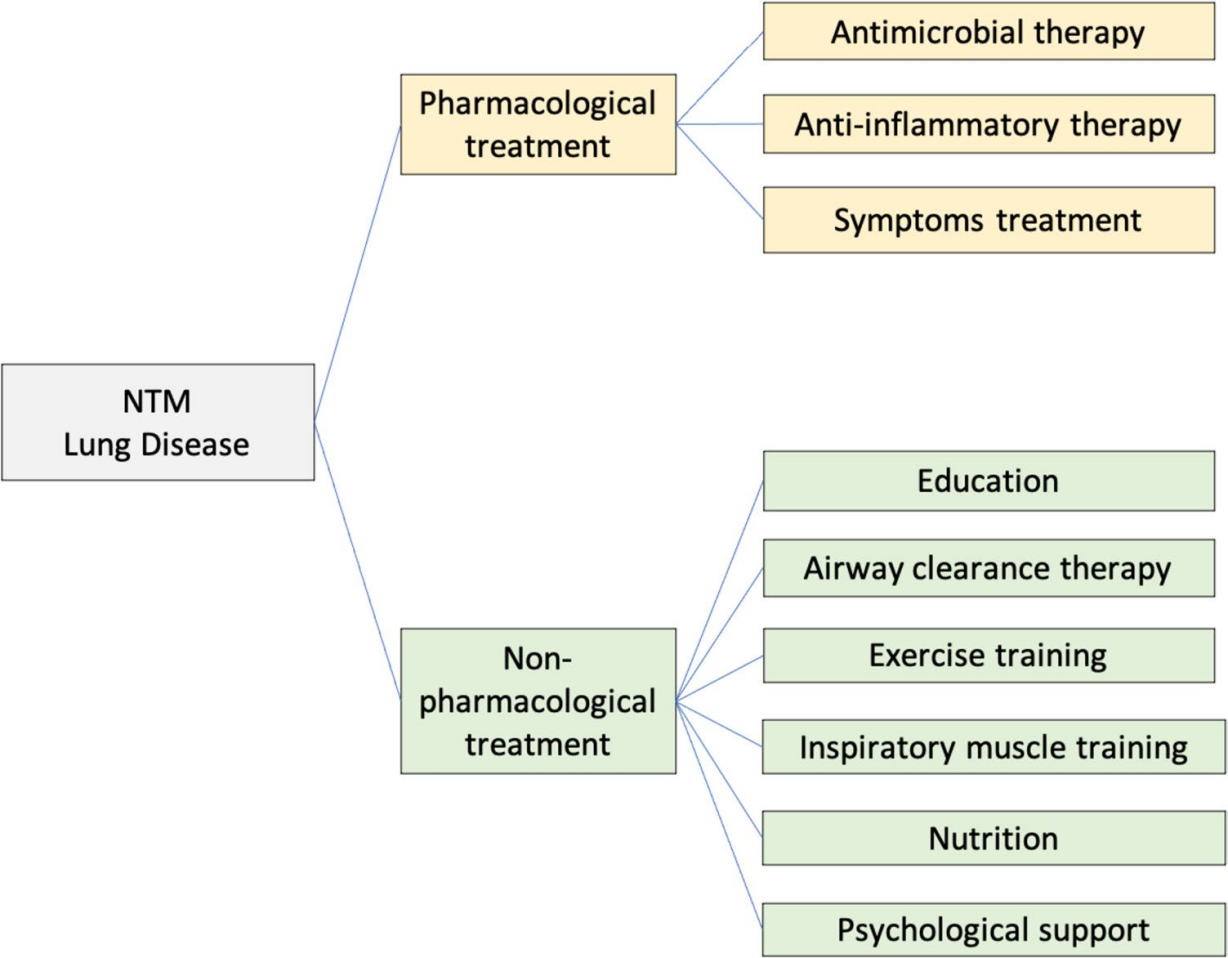
Comprehensive management of NTM pulmonary disease

Comprehensive approach to treatment of NTM-associated pulmonary disease (NTM-PD) should encompass the combination of both pharmacological and non-pharmacological treatments. This integrated approach aims at mitigating symptom severity, enhance health-related quality of life and curtail acute exacerbations. Non-pharmacological interventions include pulmonary rehabilitation (PR), nutrition support and psychological support (Fig. 1).

Pulmonary rehabilitation (PR) is defined by the American Thoracic Society (ATS) and European Respiratory Society (ERS) as a “comprehensive intervention based on a thorough patient assessment followed by patient-tailored therapies that include, but are not limited to, exercise training and educational and behavioral changes, designed to improve the physical and psychological condition of people with chronic respiratory disease and to promote the long-term adherence of health-enhancing behaviors” [26]. The benefits of PR can be summarized as follows: it reduces the need for hospitalization and alleviates symptoms of dyspnea; it enhances exercise capacity, health-related quality of life, and functional ability in daily activities; furthermore, it reinforces self-efficacy, knowledge, and collaborative self-management. Although the role and benefits of PR have been well defined in patients with COPD and bronchiectasis, there are no studies specifically assessing the role of PR and respiratory physiotherapy in patients with NTM-PD [26–29]. PR programs include educational components, airways clearance techniques, exercise training programs, and inspiratory muscle training.

Education should be provided by qualified healthcare professionals and designed to empower patients with a comprehensive understanding and effective management of NTM-PD and the underlying lung diseases. Education sessions should be tailored to address specific needs of the patients. These sessions should encompass a wide range of topics, including, but not limited to, self-care techniques, exercise training, optimal use of inhalers, airway clearance techniques, infection prevention and management, guidance on oxygen therapy, and nutritional education [27, 29].

Airways clearance techniques are deemed to be crucial to break the vicious cycle of impaired mucociliary motility, followed by microbial infection and chronic inflammation, which further impair mucociliary clearance and perpetuate the cycle. These techniques can be particularly important for patients with copious or retained secretions. Several clearance techniques have been proposed including, but not limited to, the active cycle of breathing technique, autogenic drainage, forced expiration technique, and postural drainage. However, utilization of airways clearance is observed in only around 50% of the patients, as a considerable proportion of them discontinue it within the first year of initiation. Moreover, high-quality evidence that airways clearance techniques contribute to improve the clinical outcome of NTM-PD is lacking [30].

Although studies are limited, there is increasing evidence that exercise training programs, such as cycling, treadmill workouts, walking, swimming, and resistance training, conducted over a period of 3-8 weeks, enhance exercise capacity, improve health-related quality of life, and reduce dyspnea and risk of exacerbations in patients with bronchiectasis. This intervention is particularly important for patients with diminished exercise capacity, poor health-related quality of life, and dyspnea [27, 28].

Inspiratory muscle training (IMT) is a method to train the respiratory muscle strength using a device (setting a threshold or incentive spirometry). Some studies demonstrated increased respiratory muscle strength, health-related quality of life, and exercise capacity and reduced dyspnea during daily activity after 8-week high-intensity IMT, although results were controversial in other studies [27].

Among patients affected by NTM-PD, weight loss and low BMI have been associated with disease progression, unfavorable outcomes, and increased mortality rates, even among those receiving treatment [31]. Although studies exploring the benefits of nutritional supplementation in patients with NTM-PD remain scarce, it is widely acknowledged that nutrition support and weight gain play a crucial role to help patients fight infection. As a result, careful monitoring of this aspect is warranted also in patients affected by NTM-PD. Regular consumption of small meals with high caloric content may provide advantages to patients with poor appetite, since the respiratory load during a small meal (250–500 kcal) is relatively low. Animal proteins, such as meat, fish, eggs, poultry, legumes, and dairy products, can provide essential amino acids [26, 27].

Patients with NTM-PD often experience mental health problems, that might be linked to the protracted course of the disease and the coexistence of underlying medical conditions. During the course of the disease, patients might experience repeated acute exacerbations and be repeatedly hospitalized. In addition, long-term medication is needed. Also, there might be psychological difficulties to accept the chronic nature of the disease, often posing hurdles to interpersonal relationships. In some cases, patients may experience a loss of working capacity, thus leading to economical constrains and contributing to the onset or worsening of depression, anxiety, mania, or sleep disorders. Addressing mental issues and offering timely psychological support, whenever necessary, is therefore pivotal to enhance the quality of life of the patients [32].

To coordinate this complex clinical management, a multidisciplinary approach is highly recommended. A multidisciplinary team led by a specialist physician with considerable experience with NTM, such as an infectious disease specialist or a pulmonologist, supported by a specialized nurse, is advisable. Their role should be complemented by a pharmacist, a physiotherapist, a psychologist, and a dietitian [30]. Studies concerning role and cost-effectiveness of PR and nutrition support in patients with NTM-PD remain limited and further studies, especially large RCTs, are necessary. In any case, a multidisciplinary and holistic approach is advised, encompassing both antibiotic and non-pharmacological treatment for NTM, while simultaneously addressing the management of comorbidities.

## Treatment challenges

**Fig. 2:**
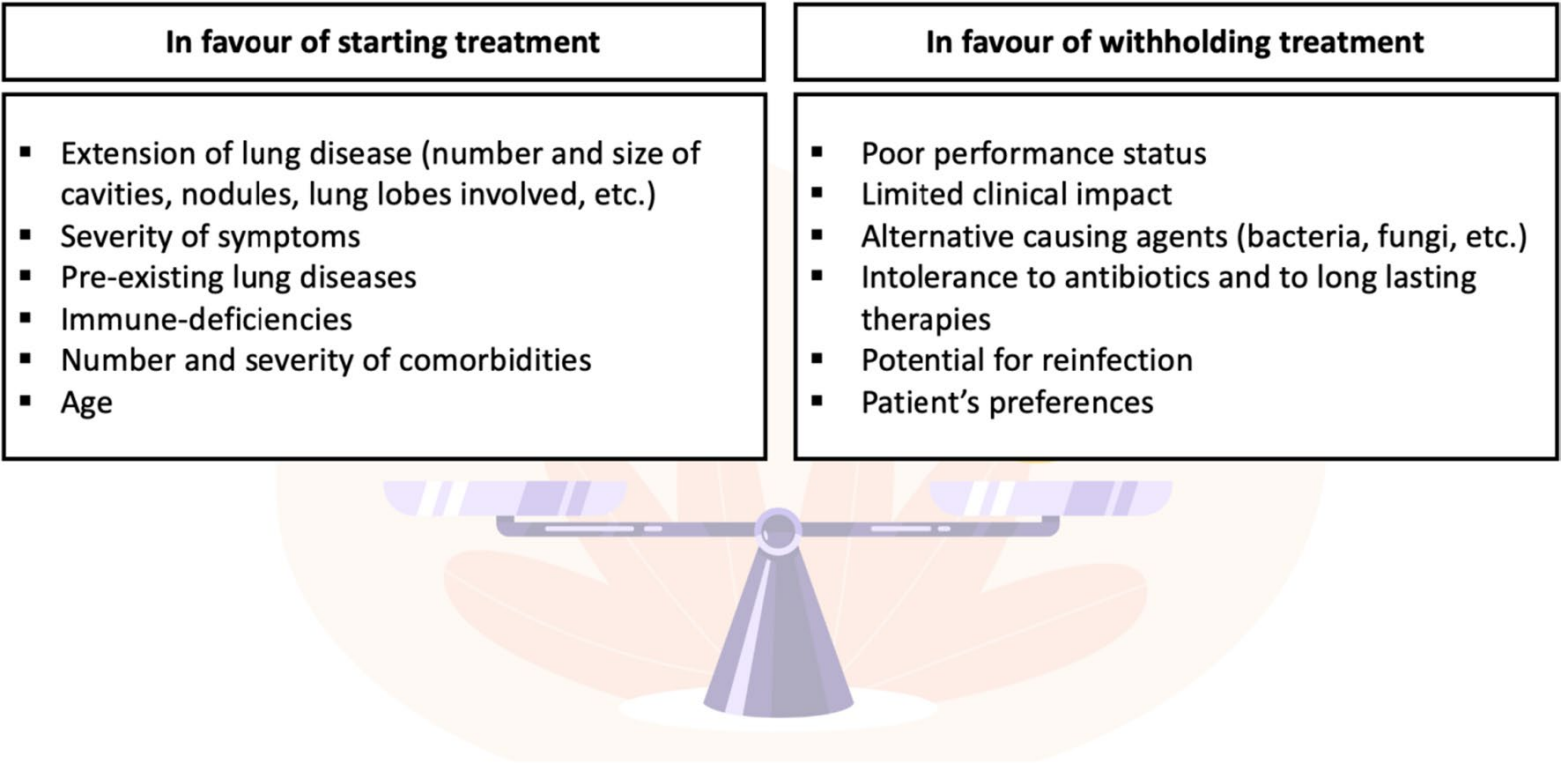
Factors to be considered when deciding if starting antimicrobial treatment in patients with NTM-PD. Background image from redgreystock on Freepik

Not all clinical forms of NTM disease require immediate treatment and, in certain cases, a strategy of "watchful waiting" may be preferred. Nonetheless, the latest international guidelines on NTM-PD recommend prioritizing treatment initiation over “watchful waiting”, especially in the presence of acid-fast bacilli in the sputum smear and/or of cavity lung disease [20]. In other cases, the decision to initiate treatment should be guided by the extent of the disease, the severity of symptoms, and the potential for exacerbating lung damage (Fig. 2).

It is important to highlight that research on the long-term clinical effects of treatment deferral is lacking. A recent study did not establish any link between the time lapse between diagnosis and treatment and patient mortality [33]. In some instances, therefore, especially when symptoms are mild or intermittent with subtle radiological changes and/or when the treatment options are limited, the potential for disease progression must be carefully balanced against the risks associated with treatment-related toxicity, the emergence of antimicrobial drug resistance, and the uncertainty surrounding the causal role of NTM (Fig. 2). In some extrapulmonary localizations (such as lymphadenitis and skin infections), surgical biopsies are needed and tissue excision is effective in treating a localized disease. The patient’s readiness and willingness to start treatment are also of paramount importance. Patient involvement in the decisional process is essential, given the prolonged treatment duration, the high incidence of side effects, and partial efficacy associated with anti-NTM treatment. Establishing a shared cure plan is imperative and its formulation hinges upon patients’ beliefs, previous experiences, intolerance, and life expectancy. Patients’ extensive information is also part of this process including the disclosures of expected treatment success rates, clinical benefits, and adverse reactions.

NTM treatment evidence indicates that the therapy outcome is largely unsatisfactory. The results should also be interpreted in terms of microbiological, radiological, and clinical success rates. Shared treatment outcomes definition have been published by van Ingen and coll [34]. It is clear that different NTM species have very different outcomes: clinical success rates in patients with NTM-PD were observed in 89.9% of those infected with *M. kansasii*, 65% with MAC and only 36.1% with *M. abscessus* (Mabs) [14]. A systematic review and meta-analysis on antibiotic therapy success rate in MAC-PD (including papers published between 1980 and 2019) showed an estimated pooled treatment success rate of 68.1% [95% confidence interval (CI) 64.7–71.4%]; the only two factors associated with better success rates were the use of macrolides and treatment duration above 12 months [35]. Another meta-analysis in patients with Mabs infections reported good outcomes in 23% participants harboring *M. abscessus* subsp. *Abscessu*s, while 84% in those with *M. abscessus* subsp. *massiliense* (OR, 0.059 [95% CI, 0.034–0.101]); sustained sputum culture conversion rates were very low and they were observed in 34% and 54% (with 20% rates in patients with refractory disease), respectively [36]. The authors concluded that “there is an urgent need to craft entirely new treatment regimens”. Similar results were reported by an individual patient data meta-analysis including 303 Mabs patients: treatment success rates were 33.0% for *M. abscessus* subsp. *abscessus* and 56.7% for *M. abscessus* subsp. *massiliense* [37]. In this context, adjunctive thoracic surgery has been evaluated in selected patients: high rates of postoperative sputum culture negative conversion (93% [95% CI, 87–97%]) and uncommon postoperative complications (17% [95% CI, 13–23%]) were recently reported [38].

## Selection of antimicrobial treatment regimens

**Fig. 3:**
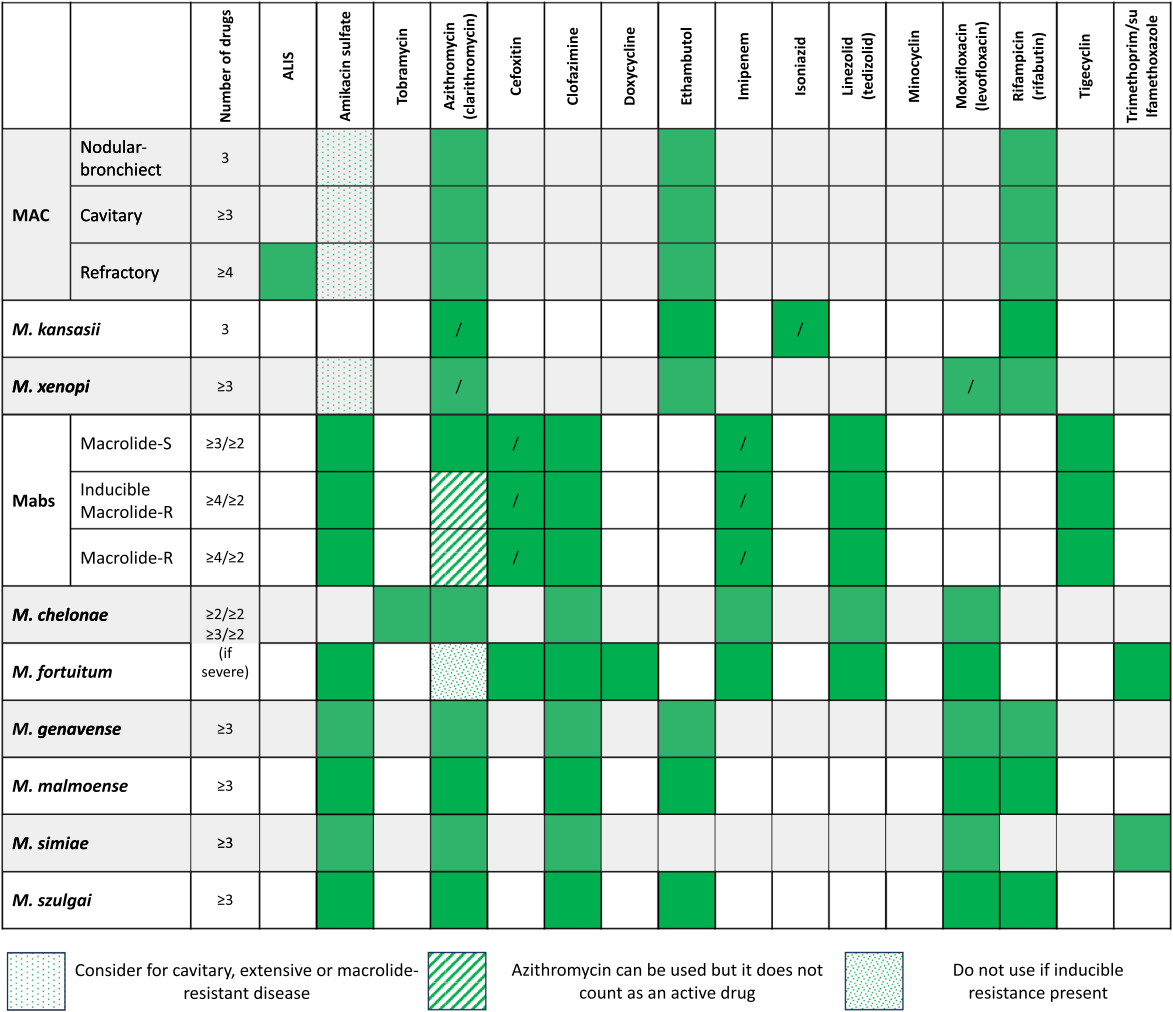
Schematic representation for the selection of first-line agents used in NTM-associated infections according to guidelines or consensus recommendations. Squares marked with “/” indicated the two drugs that can be interchanged. In the “number of drugs” column, slash signs separated the number of molecules suggested during the induction and the continuation phase. *MAC* Mycobacterium Avium Complex; *NTM* non-tuberculous mycobacteria; *M.* Mycobacterium, *Mabs Mycobacterium abscessus, ALIS* amikacin liposomal inhalation suspension

Guidelines recommend regimen selection considering the NTM species, the mycobacterial burden in the sputum, the results of drug susceptibility testing (where applicable), and the radiological features of the disease, with cavities prompting a more aggressive approach. Additionally, concomitant medications must be thoroughly reviewed due to the risk of drug-to-drug interactions and additional toxicities (especially with rifampin/rifabutin, well-known inducers of metabolizing and transporting enzymes, and with clarithromycin, an inhibitor of the P-450 enzyme system) (Fig. 3).

As already mentioned, cavities have been associated with worse prognosis than nodular-bronchiectasic forms. For instance, recent studies suggested a lower probability of achieving microbiological cure and increased risk or death in individuals with cavitary forms of the disease, particularly when cavities were larger [39, 40]. Thus, the presence of cavitary disease frequently needs a more aggressive approach, including mandatory daily of drug administration and potentially involving the use of injectable medications during the initial treatment phase. Macrolides are key drugs for the treatment of NTM: several studies identified the absence of azithromycin/clarithromycin in the treatment regimen as a significant risk factor for both microbiological and clinical failure. This was also recently confirmed by a meta-analysis [35]. Guideline-based treatments have been associated with better clinical outcomes and with the reduced selection of macrolide-resistant strains [41, 42]. ATS/IDSA guidelines suggest susceptibility-based treatment in patients with MAC, Mabs, and *M. kansasii* [20]. Additional individualization of treatment is also suggested by ATS/IDSA guidelines given the high interpatient variability in pharmacokinetics of anti-NTM drugs [43, 44]. Despite uncertain thresholds (mostly inherited from antitubercular treatment), therapeutic drug monitoring (measuring drug plasma concentrations) is suggested in selected scenarios such as malabsorption, when underdosing or drug-to-drug interactions are suspected, in case of delayed sputum conversion and in clinical conditions associated with altered drug exposure [45].

## Antimicrobials used for treating NTM-associated infections

Table 1 summarizes data on drug doses, main side effects and, pharmacokinetic data and potential optimization strategies. Each drug will be here reviewed (listed in alphabetical order) in details.

**Table 1:** Main features of the drugs used for treating NTM-associated infections

	Dosages	Dose optimization	Vd	Serum half-life (hours)	Most common adverse events	Route	Renal adjust	Liver adjust	PK targets
ALIS	590 mg once daily	Pre-medication with salbutamol?	unknown	unknown	Respiratory symptoms (dysphonia, cough, dyspnea, hemoptysis, oropharyngeal pain, bronchospasm), fatigue, diarrhoea, nausea, nephrotoxicity and ototoxicity	Nebulised (dedicated aerosol)	Avoid if severe renal insufficiency	None	Unknown
Amikacin sulfate	15 mg/Kg once daily or 25 mg/Kg 3/week	3/week	24 L	2–3	Nephrotoxicity and ototoxicity	Iv, im	10–15 mg/Kg if CrCl < 30 ml/min and further doses according to TDM	None	C_trough_ < 5 mg/L C_max_ 25–35 mg/L (7/week) or 65–80 mg/L (3/week)
Amikacin sulfate-inhaled	500 mg twice daily	Pre-medication with salbutamol	unknown	unknown	Respiratory symptoms (bronchospasm, dysphonia, sore throat, sore mouth, increased cough, wheeze and breathlessness), nephrotoxicity and ototoxicity	Nebulised (with sodium chloride 0.9%)	No specific guidance available/ Caution	None	C_trough_ < 5 mg/L
Azithromycin	250–500 mg (once daily) or 500 mg (3/week)	–	31.1 L/kg	68	Dermatological (pruritus, rash), gastrointestinal (abdominal pain, nausea, flatulence, vomiting, dyspepsia, anorexia), fatigue, CBC abnormalities (raised eosinophils, reduced lymphocytes), arthralgia, neurological (dizziness, headache, paraesthesia, dysgeusia), visual impairment and ototoxicity, QT prolongation	Oral, iv	Caution when ClCr < 10 ml/min	Avoid if severe Liver disease	C_max_ > 0.4 mg/L
Bedaquiline	400 mg daily for the first 2 weeks, followed by 200 mg 3/week	–	164 L	5.5 months (including M2)	Arthralgia, chest pain, nausea, headache, QT prolongation, increases in LFTs	Oral	None	Caution with severe liver insufficiency	Unknown
Cefoxitin	200 mg/kg/day in three divided doses (max 12 g)	Extended infusion	unknown	1	Allergy, hypotension, CBC abnormalities (leucopenia, thrombocytopaenia), increases in LFTs	iv	CrCl 10–30 ml/min: Same dose q12h CrCl < 10 ml/min: Same dose q24h	Caution with severe liver insufficiency	Unknown
Clarithromycin	500 mg twice daily	-	unknown	3–4	Gastrointestinal (abdominal pain, diarrhoea, nausea, vomiting, dyspepsia, anorexia), headache, ototoxicity, QT prolongation	Oral, iv	Avoid if severe renal disease. 50% lower dose if CRCl < 30 ml/min	Avoid if severe Liver disease	Unknown
Clofazimine	100 mg	200 mg loading dose (for 1 month)	1470 L	25–70 days	Dermatological (usually reversible pink to brownish-black skin discolouration, ichthyosis and dry skin, pruritus, rash, photosensitivity reactions), gastrointestinal (abdominal pain, nausea, vomiting, diarrhoea and weight loss), conjunctival pigmentation, QT prolongation	Oral	None	Caution with severe liver insufficiency	Unknown
Doxycycline	100 mg twice daily	Take with a full glass of water, during meals while sitting or standing	1.7 L/Kg	16–22	Dermatological (photosensitivity, skin rash), gastrointestinal (nausea, vomiting, diarrhoea, dysphagia), esophageal ulcerations, hepatotoxicity	Oral	None	Caution with severe liver insufficiency	Unknown
Ethambutol	15 mg/kg once daily or 25 mg/kg 3/week	Once daily on full stomach	76.2 L	3.3	Hyperuricaemia, gastrointestinal (nausea, vomiting), optic neuritis and red/green color blindness	Oral, iv	CrCl < 30 ml/min: 20–25 mg/kg three times weekly	Caution with severe liver insufficiency	C_max_ 2–6 mg/L
Imipenem–cilastatin	1 g/1 g twice daily	Extended infusion	0.2–0.3 L/Kg	1	Dermatological (rash and urticaria), gastrointestinal (nausea, vomiting, diarrhoea), CBC abnormalities (eosinophilia), increases in LFTs, increases in urea and/or serum creatinine concentrations, seizures	Iv, im	CrCl < 60 to ≥ 30 mL/min: 500 mg IV q8hr CrCl < 30 to ≥ 15 mL/min: 500 mg IV q12hr CrCl < 15 mL/min: 500 mg IV q12hr; not recommended unless hemodialysis instituted	Caution with severe liver insufficiency	Unknown
Isoniazid	4–6 mg/kg	Once daily on empty stomach Dose according to NAT-2 genotype	0.6 L/Kg	0.5–5 h	Liver toxicity, peripheral neuropathy (greatly reduced by pyridoxine), nausea and vomiting, drug reaction with eosinophilia syndrome, agranulocytosis and anemia, arthralgia, rhabdomyolysis	Iv, oral	None	No specific guidance available/ Caution Avoid combination with hepatotoxic drugs	C_max_ 3–5000 ng/mL
Linezolid	600 mg once daily	Consider reduction to 300 mg once daily if ADR	40–50 L	5–7	Gastrointestinal (diarrhoea, nausea, vomiting), headache, increases in LFTs, lactic acidosis, skin rash, haematological (myelosuppression), neurological (peripheral neuropathy, seizure, serotonin syndrome, optic neuropathy)	Oral, iv	None	Caution with severe liver insufficiency	C_trough_ 2–8 mg/L C_max_ 12–24 mg/L
Minocycline	100 mg twice daily	–	67–115 L	11–22	Dermatological (photosensitivity, rash), Gastrointestinal (nausea, vomiting, diarrhoea, dysphagia), Neurological (dizziness, headache)	Oral, iv	None	Caution with severe liver insufficiency	Unknown
Moxifloxacin	400 mg once daily	–	1.7–2.7 L/Kg	11.5–15.6	QT prolongation, gastrointestinal (nausea, vomiting, diarrhoea), increases in LFTs, dizziness, headache, tendon inflammation and rupture, seizures, peripheral neuropathy, worsening of myasthenia gravis	Oral	None	Caution with severe liver insufficiency	C_max_ 2.5– 4 mg/L
Rifampin	8–12 mg/kg (High dose 15–35 mg/Kg)	Once daily on empty stomach	unknown	3.3	Hepatotoxicity; Cytopenias (thrombocytopenia, hemolytic anemia, neutropenia), Flu-like Syndrome, acute renal failure (interstitial nephritis); red–orange discoloration of body fluids	Oral, iv	None	No specific guidance available/caution Avoid combination with hepatotoxic drugs	C_max_8-24 mg/L
Rifabutin	5 mg/Kg	Once daily on empty stomach	unknown	45 h	Similar to rifampicin, but hepatotoxicity more an issue if DDI increases rifabutin half-life; uveitis	Oral	None	150 mg/day if sever liver dysfunction/Caution	C_max_ 0.3–0.9 mg/L
Tedizolid	200 mg once daily	–	110 L	12	Skin rash, nausea, diarrhoea, vomiting, headache, dizziness,	Oral, iv	None	None	Unknown
Tigecycline	50 mg twice daily	Loading dose 100 mg (once), slow infusion, premedication with antihemetic drugs	unknown	27–43	Dermatological (pruritus, rash), gastrointestinal (nausea, vomiting, diarrhoea, abdominal pain, dyspepsia, anorexia), haematological (prolonged aPTT and PT), elevated LFTs, hypoglycaemia, hypoproteinaemia, neurological (dizziness, headache)	iv	None	Use with caution and at a reduced dosage (25 mg after 100 mg loading dose) if severe hepatic impairment	Unknown
Trimethoprim/sulfamethoxazole	800/160 mg twice daily	–	1.5/0.4 L/Kg	9/10	Skin rash, gastrointestinal (nausea, vomiting, diarrhoea), hyperkalaemia, headache, CBC abnormalities (anemia, leukopenia, thrombocytopenia)	Oral, iv	CrCl < 30 ml/min: 50% of usual daily dose divided q12-24 h	Avoid if severe liver impairment	Unknown

*Vd* Volume of distribution; *PK* pharmacokinetics, *ALIS* amikacin liposomal inhalation suspension; *ADR* adverse drug reactions; *CrCL* creatinine clearance; *iv* intravenous; *im* intramuscular

### Amikacin liposome inhalation suspension

Amikacin liposome inhalation suspension (ALIS) is a novel treatment that has been approved for refractory MAC-PD (FDA) or for patients with MAC-PD and limited treatment options (EMA) [93]. Data in experimental animals and in patients with CF with bacterial infections suggest a good lung penetration and a rapid uptake by alveolar macrophages [94, 95]. Besides, pharmacokinetic data confirm low amikacin systemic exposure and higher sputum concentrations. [96]. Two RCTs have been performed in patients with refractory MAC-PD. In the first one, a greater proportion of patients in the ALIS arm demonstrated at least one negative sputum culture (32% vs. 9%, *p*=0.006) and improvement in 6-minute walk test (+20.6 m vs. – 25.0 m, *p*=0.017) at Day 84; a treatment effect was mostly observed in patients without CF [97]. In the second one (CONVERT), ALIS was added to standard guideline-based therapy (GBT) in adults with amikacin-susceptible MAC lung disease and MAC-positive sputum cultures despite at least 6 months of stable GBT (224 vs. 112 participants). Culture conversion was achieved by 29.0% (ALIS + GBT) and 8.9% with GBT alone (odds ratio, 4.22) and in 13.7% vs. 4.5% of participants with clarithromycin-resistant MAC isolates (MIC >32 mg/ml) [98]. In patients who achieved culture conversion, 55.4% vs. 0% achieved sustained and durable conversion (*p*=0.0017) [99]. A French observational study reported favorable outcomes when ALIS was used in 26 patients with Mabs (with culture conversion in 54%) [100].

Data on ALIS tolerability suggest a better profile in terms of renal and ototoxicity (as compared to intravenous amikacin) but an increase in upper airways symptoms (dysphonia, cough, and dyspnea); these symptoms appeared early and were the cause of discontinuation in some patients [101]. Despite occurring in many patients, physician-guided measures (e.g., bronchodilator use, oral rinses, and/or temporary dosing adjustments) resulted in symptomatic improvement [102]. However, in the CONVERT trial serious treatment, emergent adverse events were similar between study arms (20.2% and 17.9%) [98].

The benefit of ALIS use in 331 patients from a US cohort suggested a significant reduction in respiratory disease-related (and all-cause) hospitalizations and outpatient visits were reduced in the 12 months following ALIS initiation [103].

### Amikacin sulfate (intravenous and aerosolized)

Amikacin is a semi-synthetic aminoglycoside widely used in the treatment of bacterial infections, including Gram-negative bloodstream infections in combination with other antibiotics; it is part of regimens used in the treatment of Nocardiosis, multidrug-resistant *Mycobacterium tuberculosis* (MDR-TB), and NTM diseases [20, 46, 47]. Amikacin should be considered in individuals with severe and advanced disease, such as those with fibro-cavitary forms of the disease. The uptake of aminoglycoside antibiotics by mycobacteria is an energy-dependent process and largely aerobic respiration [48, 49]. Once inside the cell, amikacin binding to the A-site located on the 16S rRNA within the bacterial 30S ribosomal subunit, causing mistranslation [50]. Furthermore, in the rapidly growing mycobacterium Mabs, amikacin has been shown to induce changes in the cell wall [51]. However, the mechanisms that induce these changes are still not completely clear, and studies have shown that they do not affect amikacin susceptibility. In mycobacteria, resistance and tolerance to amikacin, result from three main mechanisms: a) multi-site mutations within the 16S rRNA-binding site of amikacin; b) biofilm formation or metabolic changes leading to a quiescent state and reduced oxygen consumption; and c) transformation of aminoglycosides by modifying enzymes decreases antibacterial by inhibiting their binding to 16S rRNA [52–56]. Amikacin TDM has been suggested as a potential tool to optimize the drug efficacy and reduce adverse events. Target levels include low pre-dose (<5 mg/L) and high maximal concentrations (25-35 mg/L if administered daily or 65-80 mg/L if thrice weekly.

To maximize exposure by limiting the systemic toxicity of amikacin, several researchers have attempted to inhale amikacin powder by intravenous infusion [57]. Nebulized amikacin can also be considered as a replacement for of an injectable aminoglycoside, when intravenous/intramuscular administration is impractical, contraindicated, or longer term treatment with an aminoglycoside is required for the treatment of MAC pulmonary disease. Amikacin has multiple severe adverse drug reaction such as renal toxicity, neuritis of the VIII pair of nerve, cochlear and vestibular branch, neuromuscular blocks, and exanthems of various types [47]. Inhaled amikacin was associated with fewer toxic side effects as compared with the previous reports of systemic amikacin. Two adverse events are commonly reported: uncomfortable feeling in the oral cavity and hoarseness.

### Azithromycin and clarithromycin

Macrolide antibiotics inhibit protein synthesis by targeting the bacterial ribosome. They bind at the nascent peptide exit tunnel and partially occlude it. Thus, macrolides have been viewed as ‘tunnel plugs’ that stop synthesis of every protein [58]. They are considered bacteriostatic agents in clinically useful concentrations. *M. tuberculosis* is intrinsically resistant to the macrolide class of antibiotics, but the majority of clinically important NTM are sensitive to this class of antibiotics [59]: they are, therefore, the “backbone” of anti-NTM treatment.

Macrolides are unanimously considered the most important component of a treatment regimen for MAC [20]. For this infection*,* there is a clear correlation between baseline macrolide susceptibility of the causative strain and the outcome of treatment with macrolide–ethambutol–rifampicin regimens [60, 61]. Although no we need better-designed randomized trials of macrolide therapy, there is evidence that macrolide resistance is associated with a significant reduced rate of conversion of sputum conversion cultures (from positive to negative) and higher mortality [62, 63]. Optimal treatment for macrolide-resistant MAC has not been determined yet. Within the macrolide class, azithromycin is preferred over clarithromycin because of better tolerance, less drug interactions (i.e., with rifamycins), lower pill burden, once daily dosing, and equal efficacy. However, when azithromycin is not available or not tolerated, clarithromycin is an acceptable alternative. Intermittent therapy (three times a week) of macrolide containing regimens for nodular/bronchiectasic MAC pulmonary disease is included in the guidelines as an option associated with better tolerance [64]. Given the unsatisfactory efficacy of anti-NTM treatment and the importance of macrolides, this strategy is usually limited to selected patients with daily regimens being the preferred option.

Macrolides are not usually included in the initial regimen for the treatment of *M. kansasii* pulmonary disease (represented by rifampicin, ethambutol, and isoniazid). However, based on the *in vitro* activity of macrolides against *M. kansasii*, and 2 studies that demonstrated good treatment outcomes when clarithromycin was substituted for isoniazid, guidelines suggest to use either a macrolide or isoniazid for the treatment of this condition [65, 66].

Both macrolides and fluoroquinolones are active against *M. xenopi in vitro* [67]. Preliminary data from a study in France that randomized patients to receive either moxifloxacin or clarithromycin plus ethambutol and rifampicin reported no difference in the treatment success between the study arms; the final results of the trial have not been published yet [68].

In the treatment of Mabs-associated infections, macrolide susceptibility and inducible resistance are key data to create a guideline-recommended regimen [20]. While in macrolide-susceptible Mabs pulmonary disease, macrolides are recommended as first choice drugs when constitutional or inducible resistance is observed macrolides can be used but no counted as active drugs. Macrolides indeed are very active *in vitro* against Mabs strains without a functional *erm* gene, and evidence supports the use of macrolides in patients with disease caused by macrolide-susceptible Mabs [69, 70]. *Mabs* subspecies can also be relevant for their sensitivity to macrolides: the majority of Mabs subsp. *abscessus* and subsp. *bolletii* express a functional erythromycin ribosomal methylase (*erm*) gene conferring inducible resistance, while most of *Mabs* subsp *massiliense* has no functional *erm* gene. [71]. However, guidelines recommend genotype resistance test is to tailor anti-NTM regimens to the isolated mycobacterium.

Additionally, macrolides are increasingly appreciated for their anti-inflammatory and immunomodulatory effect and, on that basis, they are considered part of evidence-based treatment in and CF-related chronic respiratory infection A small study in CF patients found that azithromycin was associated with a twofold reduction in NTM isolates and that only one *M. avium* complex isolate had acquired macrolide resistance [72]. Conversely, macrolide monotherapy was recently identified as a key risk factors for the selection of macrolide-resistant MAC disease [73].

In patients with non-CF bronchiectasis, recent evidence supports that chronic administration of macrolides is related to lower rates of infectious exacerbations and, potentially, a better quality of life, especially in patients with two or more infectious exacerbations during the past year. Although these results seem to favor this strategy, prior presence of NTM-PD and possible emergence of resistance are essential issues to consider [74, 75].

### Bedaquiline

Bedaquiline inhibits the mycobacterial adenosine triphosphate (ATP) synthase, a critical enzyme responsible for the generation of ATP energy in Mycobacterium species. This unique mechanism of action offers a potential advantage in the treatment of multidrug-resistant tuberculosis, but its application in NTM-PD has not been as extensively studied [76].

Limited in vitro studies have demonstrated variable activity of bedaquiline against different NTM species. For instance, some studies have shown that bedaquiline exhibits moderate activity against MAC strains, while other NTM species might have varying susceptibilities [77].

There are scarce case reports and small observational studies describing the use of bedaquiline in patients with refractory NTM-PD. These reports typically involve patients who have failed the conventional treatment or have underlying multidrug-resistant NTM infections [78]. A small case series (*n* = 10) suggested that bedaquiline administration is associated with improved symptoms and decreased bacterial load without obtaining sustained culture conversion after 6 months of treatment [79]. A Phase II/III trial to evaluate the efficacy and safety of treatment regimens containing bedaquiline in patients with refractory MAC-PD is currently underway (trial registration: NCT04630145).

### Cefoxitin

Cefoxitin is included in multidrug regimens in cases of NTM disease due to rapidly growing mycobacteria (RGM). Its dosage ranges generally from 200 mg/kg per day up to 6-12 g daily iv, always divided into two-to-three doses. High-dose ranges are often necessary given the high MICs usually detected (see below). Cefoxitin has a half-life of 40 to 60 min when given to persons with normal renal function [80].Thus, it has been studied with administration as continuous infusion; in that case, given the most frequently detected MICs, the dose will need to be greater than 6 g in 24 h, generally 12 g [81]. Nevertheless, higher doses are accompanied to increased toxicity, especially neutropenia and thrombocytopenia [70]. Reports of *in vitro* studies show that there is great variability in susceptibility to cefoxitin among the various species and among the different areas of the world. Regarding Mabs complex, in many reports, most strains show intermediate sensitivity or resistance, while others showed full susceptibility in all strains of *M. abscessus* subspecies *massiliense* or in most strains of *M. abscessus* subspecies *bolletii* [82–84]. A nationwide study from Portugal detected *in vitro* susceptibility to cefoxitin for the majority of RGM [85].

Regardless of in vitro data, in general, there are favorable experiences in including cefoxitin in multidrug regimens to treat infections due to *M. fortuitum* and *M. abscessus*. These positive experiences include challenging clinical situations, such as bone and joint infections, pacemaker infection, meningeal infections, renal allograft, and sepsis [86–89]. It can be used with good success rates in the initial phase of combination treatment (first 4–8 weeks), in the treatment of Mabs disease as a substitute for carbapenems or in intensifying treatment regimens [70, 90, 91]. Cefoxitin can also be used safely in pediatric cases of Mabs disease [92]. Despite the available data, cefoxitin is unevenly available throughout the European Union.[93]

### Clofazimine

Clofazimine is a riminophenazine agent, recommended for leprosy and multidrug-resistant tuberculosis treatments (MDR-TB) [94, 95] .

Its 70 day half-life and high lipophilicity determine a long-term accumulation in macrophages-rich tissue [96]. Clofazimine inhibits mycobacterial respiratory chain and accumulate in membrane cells, and the effect on plasma membrane K+ transporters of T cells offers a potential immunosuppressive role [96–98]. *In vitro* clofazimine shows a bactericidal and bacteriostatic activity depending upon concentration levels after 7–14 days of administration. In murine models, clofazimine does not have an antibacterial activity on *M. tuberculosis*, but it contributes to bactericidal activity and shortening of treatment of MAC in combination therapies [98–100] and a synergistic effect of clofazimine, amikacin, and bedaquiline has been observed [101–103].

Clofazimine is orally administrated at 50 and 100 mg, but a promising study on canines on inhaled formulation shows measurable concentrations after 50 days post-dosing in lungs [104].

Although the role of clofazimine on MAC treatment is not established in international guidelines (apart from the treatment disseminated *M. chimaera* infection following cardiac surgery with cardiopulmonary bypass), in Mabs therapy, it is recommended as a part of multidrug continuation phase treatment [20, 105, 106].

A meta-analysis on 40 studies on MAC treatment estimated the success treatment rate of 56.8% in the clofazimine treatment groups; the HIV-infected subgroup patients with MAC dissemination showed a higher success rate. The immunomodulating role on macrophage and T cells could explain these results [107].

Observational and cohort study on pulmonary Mabs found a 81% symptom response and 24–50% of sputum conversion in long-term clofazimine associated treatments [108, 109]. Small case series report the efficacy and tolerability of Clofazimine in treating children with extrapulmonary disease due to Mabs.

A meta-analysis reports *in vitro* resistance of 9% and 16% for MAC and Mabs. Drug susceptibility test (DST) could be performed to guide the selection of effective treatment [113]. Moreover, DST could have an important value as conversion in sputum culture is associated with a lower clofazimine MIC [114].

Occasional adverse events (AE) have been reported. They are transient as hyperpigmentation of the skin and mucous membranes, gastrointestinal discomfort, QT interval prolongation, and hallucinations. However, according to a meta-analysis on MDR-TB, clofazimine was stopped only in 1-6% of patients, one of the lowest incidence of AE leading to discontinuation [96, 115].

### Doxycycline

Doxycycline is a tetracycline that inhibits protein synthesis by binding with the 30S and possibly the 50S ribosomal subunit of susceptible bacteria and also cause alterations in the cytoplasmic membrane. It is absorbed from the gastrointestinal tract and the average peak plasma concentration may be reduced by milk or high-fat meat. Doxycycline is lipophilic and it is widely distributed into body tissue and fluids (as synovial, pleural fluid, and bronchial secretions). For treatment of Mabs pulmonary disease, ATS/IDSA suggest performing susceptibility test to doxycycline [20]. The relationship between *in vitro* and *in vivo* results has not been established, although, in several studies, the percentage of resistance was very high [70, 116, 117]. Cantillon and colleagues showed as doxycycline has activity against *M. chimaera*, with an average MIC of 6·250 µg/mL, suggesting potential use in difficult to treat infections [118]. The recommended dose for NTM disease is 100 mg one to twice a day and the common adverse drug reactions are gastrointestinal upset, photosensitivity, and tinnitus/vertigo.

### Ethambutol

Ethambutol is a bacteriostatic anti-mycobacterial agent that inhibits the *embA, embB,* and *embC* arabinosyl-transferases of actively replicating mycobacteria, thus preventing mycobacterial cell wall formation and cell division [119, 120]. Besides *Mycobacterium tuberculosis*, ethambutol is recommended at dose of 15–25 mg/Kg as part of multidrug regimen against both cavitary and nodular MAC, *M. kansasii, M. marinum,* and *M. xenopi* NTM-PD infection [20].

Due to its good bioavailability (75–80%), ethambutol is available both in oral and intravenous formulations, and after oral administration serum peak concentrations is reached after 2 h; with a half-life of around 3.3 h in patients with normal renal function, ethambutol can be administered daily. Reduction of ethambutol absorption may occur if co-administered with aluminum salts and/or antiacids. Ethambutol undergoes partial hepatic metabolism and it is mainly excreted in the urine, needing dose adjustment in case of renal failure, while no dose change is required in case of hepatic impairment [121]. TDM should be ideally assessed between 2 and 6 h post-dose on full or empty stomach, with a desirable range of 20–60 mg/L Cmax for 25 mg/Kg dosage of ethambutol [122].

Ethambutol can be safely administered during pregnancy [123]. Dose-dependent ethambutol-induced optic neuropathy can be irreversible, and therefore, at baseline and during treatment, periodic visual acuity along with color discrimination tests should be performed [20]. Hepatotoxicity may occur especially when co-administered with other hepatotoxic drugs, such as rifamycin, pyrazinamide, and fluoroquinolones; while peripheral neuropathy and psychosis are other, less frequent, dose-dependent side effects of long-term treatment with ethambutol.

### Imipenem and meropenem

Imipenem is the most frequently studied drug for the treatment of NTM disease in this class; clinical experience exists also with meropenem [20, 124].

Imipenem is always given iv with cilastatin; its dose is variable from 0.5/0.5 g BID to four times per day or 0.75/0.75 g TID or 1/1 g BID; meropenem is used usually at the standard dose of 1 g TID. Given their iv administration, use of carbapenems is generally limited to the initial (first 1-2 months) intensive phase of treatment, but experience exists for prolonged treatment up to 6 months. Attention should be given to some potential adverse events, such as gastrointestinal disturbance (nausea, vomiting, diarrhoea), hypersensitivity (anaphylaxis, rash), central nervous system (seizures, confusion state), hepatitis, hematologic (leukopenia, anemia, thrombocytopenia).

Reports of *in vitro* studies show that there is great interspecies and geographical variability in sensitivity/resistance to imipenem among NTM. Some antibiotics, including imipenem, are unstable in culture media thus challenging the results of *in vitro* tests for predicting NTM *in vivo* susceptibility [125]. Regarding Mabs complex, in some reports, most strains show intermediate sensitivity or resistance with significant variability among subspecies [82, 126–128]. Nevertheless, there are reports of in vitro synergistic activity between clarithromycin and imipenem that may restore efficacy of the latter by reducing its MIC [129]. When comparing carbapenems activity on RGM imipenem appeared to be the most active in the family with most strains, with the exception of *M. fortuitum* and some *M. chelonae* ones that in most cases retain sensitivity to meropenem [130, 131]. Differently several slow growing NTM can present some in vitro activity for meropenem [132]. Clinically, both meropenem and imipenem have proven helpful in obtaining an effective combination regimen to treat various NTM diseases, even the most challenging [133]. Imipenem is a cornerstone of combination treatment of Mabs in the initial phase [134]. Imipenem can also be used safely in pediatric cases of Mabs disease [135].

### Isoniazid

While isoniazid is a fundamental component of antitubercular therapy, it is ineffective for treating NTM (with the notable exception of *M. kansasii)*. Prior studies have shown that MAC may be naturally resistant to isoniazid, as MICs of this drug for MAC are consistently well above that for *M. tuberculosis* and they generally exceed the concentrations achievable *in vivo* [136]*.* However, it is worth noting that in a previous randomized-controlled trial involving HIV-negative individuals with NTM-PDs caused by various NTM species, the addition of isoniazid to a macrolide-sparing regimen consisting of rifampicin plus ethambutol was associated with a lower risk of failure/relapse in the subgroup of patients infected with MAC [137]. As already mentioned, isoniazid is part of the first-line treatment of *M. kansasii.* Although no randomized-controlled trials involving patients affected by this condition has ever been conducted, observational studies have convincingly demonstrated that standard antitubercular regimens, including rifampin, ethambutol, and isoniazid yields to clinical and microbiological cure in the majority of cases [138, 139].

### Linezolid and tedizolid

Linezolid and Tedizolid are oxazolidinones, a recent class of synthetic antibiotics with a chemical structure characterized by a basic nucleus of 2-oxazolidone and an antibacterial activity due to the bind of the 50S ribosomal subunit and inhibition of the biosynthesis of bacterial proteins [140].

The oxazolidinone derivates are used in clinical practice for the treatment of multi-resistant Gram-positive bacteria (such as methicillin-resistant *Staphylococcus aureus*, vancomycin-resistant Enterococcus) and multidrug-resistant Mycobacterium tuberculosis (MDR-TB). However, oxazolidinone derivates, in particular Linezolid, are used mostly as alternative treatment for NTM infection. Despite not being recommended by guidelines linezolid is used as alternative treatment for MAC, *M. kansasii*, *M. xenopi* and is one of the preferred drugs for RGMs including macrolide-susceptible and resistant Mabs strains [20]. The Mycobacterium isolation and resistance test is important before the initiation of linezolid for NTM treatment considering that they have different resistance rates according to the species; the majority of Mabs clinical isolates showed susceptibility to linezolid, while less than 20% of MAC clinical isolates were susceptible to linezolid [141, 142].

Tedizolid is the second approved oxazolidinones derivate. Compared to Linezolid, they showed a lower *in vitro* MIC for the most common species of isolated NTM except for Mabs, but it is still promising considering the synergism with other common drugs used in clinical practice [143, 144]. Linezolid has multiple severe adverse drug reaction, such as cytopenia, peripheral neuropathy and optic neuritis, and considering the treatment length, ADRs must be investigated routinely: for example, in a study during NTM treatment, the 45% of patients had adverse event attributed to linezolid after 19.9 weeks and the 87% stopped treatment [145, 146]. Despite having a reported better tolerability, tedizolid showed similar adverse drug reactions as linezolid in a small cohort of solid organ transplant patients [147].

### Minocycline

Minocycline is a semi-synthetic derivative of tetracycline, characterized by excellent intestinal absorption (unaffected by milk or other food consumption), an extended plasma half-life and other pharmacokinetic/pharmacodynamic characteristics akin to those of doxycycline. Unlike doxycycline, however, minocycline is partially eliminated through renal filtration, requiring dose adjustment in case of severe renal failure. Historically, its application in the field of mycobacteriology has been tied with its role as a second-line treatment for leprosy. Nevertheless, doxycycline exhibits both *in vivo* and *in vitro* activity in the treatment of some non-tuberculous mycobacteria. The most substantial evidence, primarily drawn from empirical treatment or *in vitro* susceptibility data, revolves around its use as a component of treatment of *Mycobacterium marinum*, a slowly growing mycobacterium associated with indolent cutaneous infections following water exposure [148–150].

The favorable distribution within epithelial lining fluid and alveolar macrophage of minocycline, however, has positioned it also as a candidate for the treatment of NTM lung infections. In a small open-label, single-arm clinical trial involving HIV-uninfected individuals with pulmonary MAC disease, a combination of minocycline, clarithromycin, and clofazimine led to sputum culture conversion in approximately two-thirds of patients, although resistance to clarithromycin emerged in 9% [151]. Due to the lack of additional clinical evidence supporting the efficacy of minocycline for MAC treatment, the drug is not currently recommended for this indication. Regarding Mabs treatment, minocycline is listed among recommended oral antibiotics for the continuation phase [45, 134]. However, data supporting its effectiveness remain exceedingly scarce. Previous in vitro studies have suggested minocycline limited activity against circulating Mabs strains [116, 152]. Nevertheless, given the dearth of viable alternatives, the use of minocycline could be considered, particularly when guided by antimicrobial susceptibility testing.

### Moxifloxacin and levofloxacin

Fluoroquinolones (i.e., levofloxacin and moxifloxacin) exert their antimicrobial effects by inhibiting bacterial DNA gyrase and topoisomerase IV, enzymes essential for DNA replication and repair. This mechanism allows them to effectively target a wide range of pathogens, including mycobacteria causing NTM-PD [153]. Moxifloxacin is often used in combination with other antibiotics, such as macrolides and ethambutol, as part of multidrug regimens for the treatment of NTM-PD. Moxifloxacin has emerged as a potential treatment option for non-tuberculous lung disease, particularly in the context of MAC infections. A small retrospective study suggested a role for moxifloxacin in patients with refractory MAC lung disease [154].

Combination therapy has been associated with better treatment outcomes, reduced emergence of resistance, and increased bactericidal activity. Numerous studies have demonstrated the in vitro activity of fluoroquinolones against various NTM species. Levofloxacin and moxifloxacin have shown higher in vitro activity against most NTM strains, including MAC, Mabs, and *Mycobacterium kansasii*, compared to ciprofloxacin [155]. Guidelines suggest to include fluoroquinolones in first-line regimens only in the treatment of *M. xenopi.*

A retrospective study by Griffith et al. showed that the addition of a fluoroquinolone (moxifloxacin) to a multidrug regimen for the treatment of MAC-PD significantly improved sputum conversion rates in patients with MAC lung disease [45]. A prospective study by van Ingen et al. demonstrated favorable outcomes in patients treated with a combination of macrolides, ethambutol, and fluoroquinolones [34]. In a retrospective analysis including 173 patients in a tertiary referral center in South Korea, compared with the standard therapeutic regimen, clofazimine or moxifloxacin plus standard treatment regimen did not induce a higher 1-year culture conversion rate in patients with MAC pulmonary disease [156].

In another study in cavitary MAC-PD, the initial regimen replacing ethambutol with fluoroquinolones resulted in worse patient outcomes [157]. In another study, 41 patients were treated with a MXF-containing regimen because of persistent positive cultures after at least 6 months of clarithromycin-based standardized antibiotic therapy: it showed favorable treatment outcomes in about one-third of patients with persistently culture-positive MAC-PD [154].

Limited data from observational studies and case reports have suggested potential benefits of moxifloxacin in treating other NTM infections, such as Mabs and Mycobacterium kansasii.

A study by Koh et al. reported that patients receiving a multidrug regimen for Mabs pulmonary disease that included fluoroquinolone had improved sputum conversion rates and clinical outcomes compared to those who did not receive a fluoroquinolone [158].

In patients with rifampicin-resistant *M. kansasii* or intolerance to one of the first-line antibiotics, ATS/ERS/ESCMID/IDSA suggest that a fluoroquinolone (e.g., moxifloxacin) be used as part of a second-line regimen [45]. Some case reports and small observational studies have suggested the potential benefit of fluoroquinolones in treating NTM infections caused by *M. kansasii, M. xenopi*, and other less common NTM species [159]. Fluoroquinolones are associated with several adverse effects, including gastrointestinal disturbances tendinopathy, tendon rupture, peripheral neuropathy, and QT prolongation. Careful assessment of risk-benefit should be considered, especially in patients with pre-existing conditions or concomitant medications that could exacerbate these adverse effects.

### Rifamycins

Rifamycins approved for clinical use include rifampicin, rifapentine, rifabutin, and rifaximin. With the exception of rifaximin, they are part of the combination regimen for treatment of tuberculosis and NTM infections [20]. Rifamycins inhibit bacterial RNA polymerase binding specifically to the β subunit (rpoB) [160]. They are not indicated as monotherapy due to the rapid onset of resistance, resulting from mutations in rpoB gene. Since cross-resistance is incomplete, it may be appropriate to perform intra-class susceptibility testing [160, 161]. Rifamycins are cytochrome P450 enzyme system inducers and Rifampicin may also induce P-glycoprotein (P-gp) multidrug efflux transporters; therefore, patients treated with any rifamycins should be carefully evaluated for drug interactions. Rifapentine and rifabutin have longer half-life compared to rifampicin [162].

No data are currently available to establish which is the most clinically effective rifamycin in the treatment of NTM. Preference is healthcare professional dependent, considering that, in pre-clinical models, no dose-dependent difference in MAC kill nor resistance suppression has been observed [163]. Rifapentine is not routinely used in NTM infections, and it is not available in Europe. Rifapentine, co-administered with tedizolid and minocycline, showed synergism and better killing of intracellular bacteria in the intracellular hollow-fiber model system of *M. kansasii*, despite not yet shown in clinical setting [164]. Rifampicin is the most commonly used; it has high intracellular penetration ability and bactericidal effect against growing and non-growing persistent mycobacteria [165, 166]. Rifampicin is frequently used due to the lowest frequency of adverse events. The addition of ethambutol or rifampicin lowers the development of macrolide resistance [167]. The weaker effect of rifampicin for the treatment of MAC-PD could be explained by drug–drug interactions (DDIs) or by the bacteriostatic effect induced by rifampicin on slowly growing mycobacteria like MAC, instead of a bactericidal effect shown against tuberculosis. Rifampicin is also part of regimens against *M. kansasii* and *M. xenopi* (despite suboptimal evidence) but not against RGMs.

According to clinical data on high-dose tolerability, Rifampin resulted to be well tolerated when administered up to 35 mg/kg/day for patients up to 70 kg (i.e., 2400mg/day) [168–170]. Adverse events are not dose related and may derive from an immune response to the drug. The high dose does not appear to be associated with severe adverse events neither with improved clinical response rates. Therefore, the applicability of the PK/PD indices in the treatment of MAC needs to be better investigated, considering that the probability of achieving an optimal drug exposure decreases with increase of the MIC. A close monitoring, including liver function and blood cells count, is always required. Rifabutin has fewer DDIs then the other rifamycins. This is mostly evident with antiretroviral therapy, and therefore, it is used for people living with HIV to treat mycobacterial infections. High-Dose Rifabutin (600mg/day) is associated with increased number of adverse events [171]. In vitro rifabutin showed the lowest MICs against all NTM species, including MAC, M. abscessus, and M. kansasii, and showed effective activity against macrolide- and aminoglycoside-resistant NTM isolates [165]. For Mabs-PD, the guidelines suggest a susceptibility-based treatment and a multidrug regimen, including macrolide and intravenous amikacin as key drugs, without rifamycins. Mabs demonstrates ‘intrinsic’ resistance to rifampicin, but rifabutin were reported to have in vitro low MIC, synergistic (with clarithromycin, tigecycline, imipenem and cefoxitin, linezolid, and tedizolid) and additive (with clofazimine, moxifloxacin, and doxycycline) effects with other drugs against NTM species, no antagonism, bactericidal activity, high cellular penetration, suitable concentrations in human lung tissue, and reduced DDIs, but adverse reactions are often reported [172, 173]. Recent data challenge the utility of rifamycins in the treatment of NTM (maybe with the exception of *M. kansasii* combination therapy): beside the well-known PK interaction that may significantly reduce macrolides’ concentrations, in a hollow-fiber model, rifampicin did not potentiate the anti-mycobacterial effect to the 2-drug therapy or did not suppress the emergence resistance [174, 175].

### Tigecycline

Tigecycline (TGC) is the first glycylcycline antibiotic to be approved by the U.S. Food and Drug Administration. The drug overcomes the two major resistance mechanisms of tetracycline: drug-specific efflux pump acquisition and ribosomal protection. TGC has demonstrated activity against rapidly growing mycobacteria (RGM), such as *M. chelonae*, Mabs, and *M. fortuitum* [176, 177]. In a comparative *in vitro* study that included 72 isolates of RGM, tigecycline MICs were ≤1 mg/L for all tested tetracycline susceptible and tetracycline-resistant isolates of Mabs, *M. chelonae,* and *M. fortuitum* [116]. In 2009, investigators from Spain determined the antimicrobial susceptibility of RGM (including *M. fortuitum, M. chelonae*, Mabs, and others) using the Etest method (a non-CLSI-approved susceptibility testing method for these species) in 54 clinical isolates. The authors reported that all strains were inhibited by tigecycline at very low MICs [178].

The usual dose used in clinical practice of TGC is 25–50 mg once or twice intravenous infusion (iv) per day but most experts recommend once daily dosing of TGC due to the high rate of drug-related adverse reactions associated with twice daily dosing (i.e., nausea, vomiting, hepatitis, and pancreatitis). Furthermore, administration of TGC via iv decreased patient compliance, especially due to its long course.

The majority of *M. chelonae* isolates are sensitive to clarithromycin, tobramycin, and linezolid. TGC may be a useful agent combined with other active drugs to treat *M. chelonae* infection, although further large-scale randomized studies would need to be performed to determine its true effectiveness [179]. *M. fortuitum* was reported susceptible to multiple drugs except for macrolides indeed most isolates have an active *erm* gene [71]. The antibiotics resistance spectrum varies with different geographic locations or hospital administration situation. The role of *in vitro* drug susceptibility testing may be nevertheless important in the management of NTM-related diseases [180].

Wallace and coll. demonstrated that tigecycline-containing regimens for salvage treatment of RGM infections improve clinical conditions; in this setting, tigecycline could be clinically beneficial as part of a multidrug treatment strategy, especially against RGM species causing serious disease, before susceptibilities are available [181].

### Trimethoprim–sulfamethoxazole

Guidelines and consensus reports suggest the use of trimethoprim–sulfamethoxazole for limited NTM cases (resistance to first-line drug, rare species as *M. fortuitum* and *M. simiae*) [20, 182]. The recommended dose is 800/160 mg twice daily. In several studies, NTM showed high levels of resistance: 95.8% for Mabs (Mabs subsp. *massiliense* was reported to be between 0 and 97% susceptible) and 64.3% for *M. kansasii* [82, 183, 184]. No data from clinical trials are available, but case reports suggest the use for rare or difficult to treat NTM diseases [185].

### New drugs with preliminary available data

New tetracycline antibiotics have shown efficacy in treating RGM infections [186–188]. Eravacycline has demonstrated more activity against RGM than SGM in *in vivo* studies [189, 190]. Omadacycline has displayed strong activity in multiple *in vitro* and *in vivo* studies, making it a potential candidate for clinical use, particularly against Mabs, as supported by evidence from *in vitro* studies and case series [191–194]. Although reduced activity against SGM has been observed in some studies, omadacycline has shown lower MIC values against *M. kansasii* and MAC [195, 196].

Oritavancin, a novel lipoglycopeptide, has shown *in vitro* bactericidal activity against Mabs, with the ability to reduce mycobacterial load in the lungs when used alone or in combination with other antibiotics [197].

Fidaxomicin, a semi-synthetic macrolide, has high *in vitro* activity against Mabs, MAC, *M. fortuitum, M. kansasii, and M. parascrofulaceum*, without inducing resistance to macrolides in Mabs complex, unlike clarithromycin [198].

Delamanid, a new antibiotic derived from nitro-dihydro-imidazooxazole, inhibits the synthesis of mycobacterial cell wall. Two studies showed only moderate activity against certain species of SGM, with lower MIC values for *M. kansasii* [199, 200]. Pretomanid, a bicyclic nitroimidazole, has shown promising results in reducing the load of Mabs in the lungs and spleen of mice, although previous *in vitro* studies reported high MICs for most NTM except for *M. kansasii* [201, 202].

The presence of the β-lactamase BlaMab limits the activity of β-lactam antibiotics against Mabs [203]. However, novel β-lactamase inhibitors, such as diazabicyclooctane and cyclic boronate, have been shown to inhibit BlaMab, unlike clavulanate, tazobactam, and sulbactam. Avibactam has demonstrated its ability to inactivate BlaMab and increase the efficacy of imipenem, piperacillin, and tebipenem in in vivo and *in vitro* studies [204]. Other β-lactamase inhibitors, including relebactam, nacubactam, zidebactam, and vaborbactam, have also shown potential in increasing the activity of carbapenems and other β-lactams in both *in vivo* and *in vitro* studies [205–209].

### Phage therapy

Because of the unsatisfactory treatment outcomes and resistance issues, phage therapy has been considered in hard-to-treat NTM infections (mostly Mabs). Despite anecdotal use in drug-resistant bacterial infections, several aspects of phage therapy remain unclear (such as suitable types of infections and pathogens, routes, dosage, frequency of administration, interactions with antibiotics, and pharmacokinetics). The repertoire of therapeutically useful phages is small, and mostly limited to phages isolated on *M. smegmatis* with only few phages isolated directly on any strain of Mabs [210]. There is great variation in phage susceptibilities among Mabs clinical isolates: while rough strains have at least one active phage, no one has so far been identified for smooth colony morphotypes (approximately 40% of Mabs isolates) [55, 211]. After two effective phage treatment for Mabs had been reported in 2019 and 2022, a recent case series was published [210, 212, 213]. In the latter one, *Mycobacterium* isolates from 200 culture-positive patients with symptomatic disease were screened for phage susceptibilities. One or more lytic phages were identified for 55 isolates. Phages were administered intravenously, by aerosolization, or both to 20 patients on a compassionate use basis. While no adverse reactions attributed to therapy were seen, favorable clinical or microbiological responses were observed in 11/20 patients. Neutralizing antibodies were identified in serum after initiation of phage delivery intravenously in 8 patients, potentially contributing to lack of treatment response in 4 cases, but were not consistently associated with unfavorable responses in others. Eleven patients were treated with only a single phage, and no phage resistance was observed [213].

## Treatment of underlying diseases

Immunotherapies for infectious diseases are generally defined as host-directed therapies (HDT) which are interventions with an impact on immunity (innate or adaptive) or intracellular immune responses to microbial pathogens with the aim to stimulate the immune response against the pathogen or to prevent the tissue damage mediated by the immune response directed to the pathogen [214]. HDTs may offer advantages compared to the standardized antibiotic therapy, because can be effective against both drug-resistant and drug-susceptible pathogens and likely against potentially dormant mycobacteria. Moreover, HDTs may synergize with, or shorten antibiotic treatment by targeting different pathways, thus reducing toxicity without affecting the treatment efficacy.

NTM are recognized by host innate immune cells which have the ability to promote the intracellular mycobacterial killing [215]. However, the mycobacteria have generated strategies to persist inside the host cells reducing the phagosome acidification and maturation, escaping from the phagosomes into the nutrient-rich cytosol, blocking the cell autophagy, reducing the antigen presentation, and impairing T-cell immunity. Several therapeutical approaches have been tried to overcome these obstacles. Some examples are reported as imatinib that promotes phagosomal acidification and autophagic flux in *M. marinum*, GM-CSF that increases phagocytosis and auto-phagolysosome fusion in *M. avium*, IL-2 or IFN-γ that promote TH-1 immunity against *M. avium*, cysteamine that reduces in vitro replication of Mabs and can synergize *in vitro* with amikacin to reduce the pathogen growth. Interestingly, cysteamine can also reduce inflammatory response, as also shown in viral infections [216–224].

Recently, analyzing encounter-level data from the US Cystic Fibrosis Foundation Patient Registry from 2011 to 2018, it has been shown that in CF patients, ivacaftor, a drug modulating the transmembrane conductance regulator (CFTR), is associated with a decreased risk of NTM [225]. Among the different effects of the drug, ivacaftor favors mucus clearance and pulmonary function, leading to a reduced risk of pulmonary NTM.

## Conclusions

NTM are a group of very heterogeneous mycobacteria that can cause a wide range of infections in humans and whose incidence has increased in recent years. The treatment of NTM infections is challenging, because currently available regimens require very long durations and have a high incidence of adverse events with unsatisfactory microbiological, clinical, and radiological outcomes. New drugs as well as treatment strategies tested in randomized and controlled studies are urgently needed. We discussed the most recent evidence on these topics and reported the available data on drugs used for treating NTM infections: optimization strategies as well as potential therapeutic alternatives are proposed to optimize current treatment options.

### Supplementary Information

Below is the link to the electronic supplementary material.

Supplementary file1 (DOCX 19 KB)
