# Supplementary material for: Drugs for treating infections caused by non-tubercular mycobacteria: a narrative review from the study group on mycobacteria of the Italian Society of Infectious Diseases and Tropical Medicine
Source: Infection. 2024 Feb 8;52(3):737–65. doi: 10.1007/s15010-024-02183-3 (PMC11142973; doi:10.1007/s15010-024-02183-3)
Supplement: Supplementary file 1 — Supplementary file1 (DOCX 19 KB) [file 15010_2024_2183_MOESM1_ESM.docx]

**Supplementary table 1. Epidemiological and microbiological characteristics of NTM infection in different countries.**

| **Author** | **Enrollment period** | **Country** | **Type of Study** | **Age included** | **Localization** | **Most common species** | **Prevalence of NTM** | **Incidence of NTM** | **Increase of incidence** |
| --- | --- | --- | --- | --- | --- | --- | --- | --- | --- |
| Aliano D. et al [15] | 2000-2017 | Australia | Observational | 0-14 years old | Extrapulmonary | M. fortuitum; M. abscessus; M. avium; M. intracellulare | Not defined | Not defined | Not defined |
| Blakney R. A. et al [28] | 2005-2019 | Hawaii | Observational | ≥18 years old | Pulmonary Infection | Not defined | Not defined | Overall 44.8/100.000 | Not defined |
| Smith SG et al [3] | 2006-2010 | United States of America | Observational | No restriction | Pulmonary Infection (79.4%); extrapulmonary (20.6%) | M. avium complex(48.3%); M. gordonae (16.5%); M. abscessus complex(17.9%) | 14.9/100.000 (2006); 12.2/100.000 (2010) | 15.9/100.000 (average) | Not defined |
| Lin C. et al [4] | 2007-2011 | Hawaii | Observational | No restriction defined | Respiratory specimen | MAC (20%), M. fortuitum (14.3%), M. gordonae (5.7%) | Overall 106/100.000 | Increased isolation from 0,5% (2007) to 11.3% (2011) and incidence from 2 /100.000(2007) to 48/100.000 (2011) | Increased |
| Henkle E. et al [5] | 2007-2012 | Unites States of America | Observational | No restriction defined | Extrapulmonary | MAC (50%), RGM (38.6%) | Not defined | Average 1.5/100.000 | Not increased |
| Jones MM et al [6] | 2008-2012 | United States of America | Observational | No restrition defined (Veteran cohort) | Pulmonary and extrapulmonary | MAC (69.91%), RGM (16.8%) | Not defined | Average 12.6/100.000 | Not defined |
| Donohue MJ et al [22] | 2008-2013 | United States of America | Observational | No restriction | No restriction | Not defined | Average 12.6/100.000 | Increased incidence from 8.7 /100.000 (2008) up to 13.9/100.000 (2013) | Increased |
| Winthrop K.L. et al [23] | 2008-2015 | United States of America | Observational | No restriction defined | Pulmonary Disease | Not defined | 6.78/100.000 in 2008 up to 11.7/100.000 in 2015 | Increased incidence from 3.13/100.000 in 2008 up to 4.73/100.000 in 2015 | Increased |
| Garcia V.C. et al [29] | 2011-2017 | United States of America | Observational | ≥12 years old | No restriction | Not defined | M. abscessus group (45.4%), M. gordonae (19.9%), MAC (8.9%) | Not defined | Not defined |
| Lopez-Luis B.A. [7] | 2001-2017 | Mexico | Observational | ≥18 years old | Pulmonary (41.7%), bloodstream and disseminated disease (46%), SSTI (12.3%) | MAC was the most common SGM, and M. chelonae was the most frequent RGM during first 5 years, than M. fortuitum and M. abscessus. | Not defined | incidence 2001-2011 was 0.6 /1000 admission for SGM and 0.3/1000 admission for RGM; incidence 2012-2017 was 1.9/1000 admission for SGM and 1.1/1000 admission for RGM. | Increased |
| Pedrero S. et al[30] | 1997-2016 | Spain | Observational | ≥18 years old | Pulmonary disease | M. kansasii (83.8%), MAC (13.1%) | Not defined | Incidence Rate ratios (IRR) period 1997-2006 0.939 (95%CI 0.899-0.980); IRR period 2007-2017 0.920(95%CI 0.849-0.997).  Highest incidence in 2000 with 10.6/100.000; incidence in 2016 1.8/100.000 | Not Increased |
| Hermansen T.S. et al [8] | 1991-2015 | Denmark | Observational | No restriction | No restriction | MAC, M. gordonae, M. abscessus/chelonae | Not defined | 1.20/100.000 for definite NTM disease; 0.49/100.000 for possible NTM disease, 0.88/100.000 for NTM colonization. Average Pulmonary incidence 1.78/100.000, average non-pulmonary incidence 0.69/100.000. patients aged >15 years Had incidence of pulmonary and non-pulmonary of 2.14 and 0.38/100.000 respectively; instead patients with less than 15 years old had pulmonary and non-pulmonary incidence of 0.20 and 2.0/100.000 year, respectively. | Not increased |
| Ringshausen F.C. et al.[13] | 2009-2014 | Germany | Observational | No restriction | Pulmonary Disease | Not defined | Not defined | from 2009 to 2014 incidence increased from 2.3 to 3.3 /100.000 | Increased |
| Panagiotou M. et al[14] | 2007-2013 | Greece | Observational | No restriction | Pulmonary Disease | MAC (27%), M. gordonae (13.9%), M. fortuitum (12.2%) | Not defined | Overall NTM pulmonary infection and NTM-disease 18.9 and 8.8/100.000, respectively | Not defined |
| Ringshausen F.C. et al.[24] | 2005-2011 | Germany | Observational | No restriction | Pulmonary Disease | Not defined | Not defined | average 0.91/100.000, from 0.73 in 2005 to 1.09/100.000 in 2011 | Average increase of 5.9% (95%CI 4.7-7.) per year |
| Rindi L and Garzelli C.[10] | 2004-2014 | Italy | Observational | No restriction defined | Pulmonary Disease and extrapulmonary | MAC (55.8%), M. gordonae (11.6%) | Not defined | increased from 5 isolates in 2004 to 29 in 2014 | Increased |
| Prato DB. et al [11] | 2016 | Italy | Observational | No restriction defined | Pulmonary Disease | MAC, M. xenopi, M. kansasii, M. abscessus | Not defined | Not defined | Not defined |
| Dahl NV. et al [31] | 2011-2021 | Denmark | Observational | No restriction defined | Pulmonary Disease and extrapulmonary | Not defined | Not defined | from 1.3/100.000 in (2013) up to 2.5/100.000 in 2021 | Increased |
| Jarchow-MacDonald et al [32] | 2011-2019 | United Kingdom | Observational | No restriction defined | Pulmonary Disease and extrapulmonary | M. avium (45.2%), M. intracellulare (19.4%), M. abscessus complex (11.7%) | Not defined | increased from 3.4/100.000 in 2011 up to 6.5/100.000 in 2019 | Increased |
| Dakic I. et al [16] | 2010-2015 | Serbia | Observational | No restriction defined | Pulmonary Disease | Most common isolated M. xenopi (17.3%), M. gordonae (12.9%), M. fortuitum (11.3%); most common associated with NTM-PD was M. xenopi (28.6%) but decrease during observation, RGM (27.8%) increase during observation | Not defined | Isolation frequency incidence 0.89/100.000 in 2010 up to 1.63/100.000 in 2015; NTM-PD incidence from 0.18/100.000 in 2010 up to 0.48/100.000 in 2015. | Increased |
| Schildkraut J.A. et al [33] | 2016 | Europe and Japan | Observational /estimated prevalence | No restriction defined | Pulmonary Disease | Not defined | Not defined | 6.2/100.000 in Europe; 24.9/100.000 in Japan | Not defined |
| Zhou L. et al [34] | 2000-2019 | China | Sistematic Review and Meta-analysis | No restriction defined | Isolation rate | M. avium and M. gordonae most common reported in HIV, M. marinum and M. abscessus most common in bacteriemia; M. avium and M abscessus most common in pulmonary infection; M. chelonae and M. marinum most common in SSTI; m. fortuitum and M. abscessus in bone and joint infection. | Not defined | Not defined | Not defined |
| Lee M. et al [12] | Not defined | Taiwan | Sistematic Review | Not applicable | Not applicable | MAC, M. abscessus complex and M. kansasii are the most common species | Not defined | Increased from 3.4/100.000 in 2000 up to 13/100.000 in 2012; 21.9/100.000 in northen taiwan in 2014 and 106.4/100.000 in southern taiwan. | Increased |
| Kim JY et al [35]] | 2010-2021 | South Korea | Observational | No restriction defined | No restriction | Not defined | Not defined | increased from 11.4/100.000 in 2010 up to 56.7/100.000 in 2021 | Increased |
| Huang J. et al [36] | 2013-2018 | China | Observational | No restrition | No restriction | Not defined | considering patients hospitalized for micobacterial disease, NTM increased from 15.6% in 2013 up to 46.1% in 2018. | Not defined | Not defined |
| Ide S. et al [13] | 2001-2010 | Japan | Observational | No restriction | Pulmonary Disease | M. intracellulare (44.3%), M. avium (42.6%) | Not defined | 4.6/100.000 in 2001; 10.1/100.000 in 2009 | Increased |
| Okoi C. et al [14] | 1940-2016 | Sub-Saharan Africa | Sistematic Review and Meta-analysis | No restriction | Pulmonary Samples | MAC predominated (27.7%) | Not defined | Not defined | Not defined |
